# Farmers’ preferred tree species and their potential carbon stocks in southern Burkina Faso: Implications for biocarbon initiatives

**DOI:** 10.1371/journal.pone.0199488

**Published:** 2018-12-18

**Authors:** Kangbéni Dimobe, Jérôme Ebagnerin Tondoh, John C. Weber, Jules Bayala, Korotimi Ouédraogo, Karen Greenough

**Affiliations:** 1 West African Science Service Centre for Climate Change and Adapted Land Use (WASCAL) Competence Center, Ouagadougou, Burkina Faso; 2 University Ouaga I Pr Joseph Ki-Zerbo, UFR/SVT, Laboratory of Plant Biology and Ecology, Ouagadougou, Burkina Faso; 3 UFR des Sciences de la Nature, Université Nangui Abrogoua, Abidjan, Côte d’Ivoire; 4 World Agroforestry Centre (ICRAF), Lima, Peru; 5 World Agroforestry Centre (ICRAF), West and Central Africa, Sahel Node, Bamako, Mali; 6 Université du Faso (UFA), Ouagadougou, Burkina Faso; Fred Hutchinson Cancer Research Center, UNITED STATES

## Abstract

The success of terrestrial carbon sequestration projects for rural development in sub-Saharan Africa lies in the (i) involvement of local populations in the selection of woody species, which represent the biological assets they use to meet their daily needs, and (ii) information about the potential of these species to store carbon. Although the latter is a key prerequisite, there is very little information available. To help fill this gap, the present study was undertaken in four pilot villages (Kou, Dao, Vrassan and Cassou) in Ziro Province, south-central Burkina Faso. The objective was to determine carbon storage potential for top-priority woody species preferred by local smallholders. We used (i) participatory rural appraisal consisting of group discussions and key informant interviews to identify priority species and functions, and (ii) landscape assessment of carbon stocks in the preferred woody species. Results revealed 79 priority tree and shrub species grouped into six functions, of which medicine, food and income emerge as the most important ones for the communities. For these functions, smallholders overwhelmingly listed *Vitellaria paradoxa*, *Parkia biglobosa*, *Afzelia africana*, *Adansonia digitata*, *Detarium microcarpum*, and *Lannea microcarpa* among the most important tree species. Among the preferred woody species in Cassou and Kou, the highest quantity of carbon was stored by *V*. *paradoxa* (1180 ±209 kg C ha^-1^ to 2089±522 kg C ha^-1^) and the lowest by *Grewia bicolor* (5±1.2 kg C ha^-1^). The potential carbon stored by the preferred tree communities was estimated at 587.9 Mg C ha^-1^ (95% CI: 456.7; 719.1 Mg C ha^-1^) in Kou and256.8 Mg C ha^-1^ (95% CI: 67.6; 324.4 Mg C ha^-1^) in Cassou. The study showed that the species that farmers preferred most stored more carbon than species that were less preferred.

## Introduction

In the West African Sahelian and Sudanian agro-ecological zones, parkland agroforests are socio-ecological systems that integrate trees, crops and livestock. They play key roles in the functioning of agro-ecological landscapes, delivering essential goods and many ecosystem services that sustain smallholder farmers and pastoralist livelihoods [1–8]. Delivery of provisioning ecosystem services, including food, fodder and fuel wood, contributes greatly to rural communities’ daily needs. Income from these products helps to improve livelihoods and build resilient socio-ecological systems in the face of ongoing climate change and variability [9]. These integrated tree-crop-livestock systems are subject to severe degradation through deforestation due to both climate change [10] and unsustainable land management practices, such as overgrazing and wood cutting [11,12]. This poses immediate threats to smallholders’ sustainability and coping abilities in confronting the adverse impacts of climate change. Land degradation leads to a reduction of vegetation cover, species richness and abundance. The corollary is an increase in soil erosion, depleting soil nutrients, including soil organic carbon. Thus, the low standing biomass of degraded land is associated with low soil carbon and diminished productive properties, weakening the resilience of farming systems and that of people making their living from these systems [13].

Restoring agro-ecological functions for increased productivity and resilience requires climate- smart land uses [14,15]. By encompassing a set of land cover options and management practices that increase greenhouse gas (CO_2_) absorption and biocarbon stocks, these land uses can also help mitigate climate change by reducing overall concentration of CO_2_ in the atmosphere [13]. Biocarbon projects are among the options which have been promoted in the framework of the Kyoto Protocol and more recently through reducing emissions from deforestation and forest degradation (REDD+) initiatives. The latter intend to contribute to local development by generating carbon-based incomes for smallholders through carbon markets. Co-benefits include supporting ecosystem services like improved soil fertility, and provisioning services like food and income [13,16,17]. Biocarbon projects are diverse in scope, and a large share of World Bank Biocarbon Fund investments go into environmental restoration of degraded lands (50.5%), fuelwood production (23%) and timber production (20%) [15].

The effectiveness of biocarbon initiatives to improve smallholders’ livelihoods through carbon finance has been challenged, particularly in the Sahel [18], because of many bottlenecks, including (i) the dearth of empirical data on the potential of farmers’ preferred tree species to store carbon, (ii) the long time it takes for biocarbon projects to become economically viable and profitable, (iii) the complexity of access to carbon markets, (iv) the uncertainty about future climate, and (v) low carbon prices in international markets. Farmer-managed natural regeneration (FMNR), a land management practice many Sahelian farmers use to rehabilitate their degraded lands, could be the foundation for biocarbon initiatives [19,20]. However, the data and knowledge gap (carbon storage potential) must be bridged in a participatory way before best-fit options for Sahelian biocarbon initiatives may be scaled-up.

Aboveground plant biomass (AGB) is defined as the amount of organic matter in living and dead plant material above the soil surface [21] and serves as indicator for carbon stock in that compartment. Measuring AGB in terrestrial vegetation is needful to implementing land-based climate mitigation strategies [22]. AGB can be estimated using biomass expansion factors or allometric equations. On large scale, satellite- or aircraft-based remote sensing methods are coupled with field measurements. Allometric equations are fundamental tool for non-destructive estimation of biomass in woody vegetation, which further can be up-scaled to stand level, thus contributing to accurate accounting of stock and flux of biomass and carbon in terrestrial ecosystems [23]. These equations express tree biomass as a function of easy-to-measure parameters such as diameter at breast height (DBH), height or wood density or a combination of these parameters [24–26]. The equations can be developed for estimating biomass of specific species, or a mix of species from a region. In Burkina Faso, allometric equations have been developed for some economically important tree species, for example *Vitellaria paradoxa* [8], *Combretum glutinosum* and *Terminalia laxiflora* [27], *Bombax costatum* [28], *Jatropha curcas* [29] and some 11-native species [30]. But protected areas are mostly multi-species and require mixed species equations developed with data that covers the diversity of the trees to be measured. These equations are lacking in Burkina Faso, hence general equations such as those reported in Chave et al. [23,26] are commonly used for estimation of biomass for general purposes.

The present study is mainly guided by the lack of information on farmer’s preferences for tree functions and the priority plant species for these functions in Burkina Faso. Understanding which species are most important for local people and the reason they are important to them is an asset to the development of participatory projects for sustainable management and cultivation of the species that local people are likely to conserve [31,32]. Hence, the aim of this study was to determine carbon storage potential for top-priority woody species preferred by local smallholders. Specific objectives were to (i) determine farmer’s preferences for tree function and priority woody species in Burkina Faso and (ii) estimate carbon stocks of woody species that local communities deemed top-priority or very useful. We hypothesized that farmer’s preferences for tree functions and woody species vary significantly among villages and by gender, that farmers retain or plant on their farms species that provide them with food and medicine, and that preferred woody species store the largest percentage of carbon in the study area. This work was carried out within the framework of the Building Biocarbon and Rural Development in West Africa (BIODEV) project implemented in Burkina Faso, Guinea-Conakry, Mali and Sierra Leone. The overall goal of the project was to demonstrate the multiple developmental and environmental benefits that result from a high value biocarbon approach to climate change and variability in large landscapes. The current study falls under “Agroforestry and farm interventions” that aimed at increasing the adoption of agroforestry and other carbon-enriching farm practices that meet beneficiaries’ priority needs and address climate change issues.

## Materials and methods

### Study area

**Fig 1:**
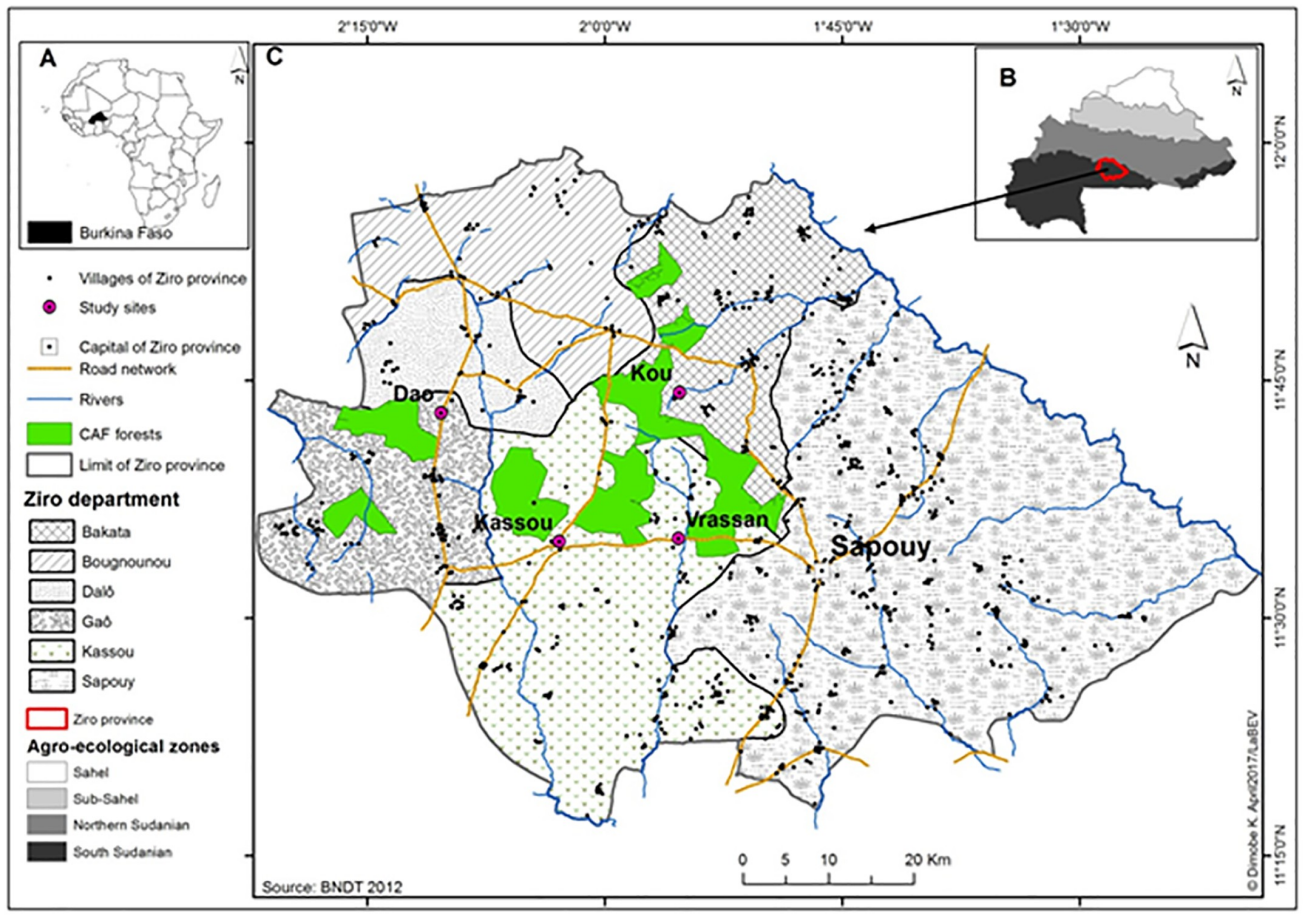
Location of the study area. (A) the location of Burkina Faso in Africa, (B) the position of province of Ziro in Burkina Faso, and (C) the province of Ziro with study sites. Study sites are indicated by circles while polygons show the limits of managed forest under the CAF scheme.

The study sites were located in Kou, Dao, Vrassan and Cassou (Fig 1), located in Ziro Province (11°16’N to 11°45’N and 2°10’W to 1°48’W) Burkina Faso. These villages were selected because local populations have close interactions with the protected Cassou Forest [12,33].

The Ziro province covers 5,291 km² and is characterized by a low-relief topography with a mean altitude of 300 masl. Phytogeographically, this province is located within the south-Sudanian agro-ecological zone [34]. Rainfall follows a unimodal pattern that lasts for six months (May through October), and mean temperatures is 30°C, with higher temperatures (40°C) during the dry season. Twenty-five villages surround the 30,000 ha of the Cassou forest, comprising a mixture of mostly dry natural forest and tree savannah vegetation types. The main soil types are silt-clay cambisols, sandy lixisols, and loamy ferric luvisols. Agroforestry parklands dominated the agricultural landscape with intermittent fragmented dry forests.

The province has one of the highest average rural population densities in the country, with an estimated 30 inhabitants per km² [35,36]. The dominant crop production systems are cereals rotated with tubers, and animal husbandry.

Supervised by a private organization, *Chantiers d’Aménagements Forestiers* (CAF, Community Forest Management), local populations jointly implement a management plan for the Cassou forest. The CAFs were created by the government in this region to ensure a sustainable supply of commercial fuel wood, construction poles, and other forest products to the nearby cities of Ouagadougou and Koudougou [33], and thereby contribute to local community livelihoods. The CAFs’ primary challenge lies in the sustainable management of the forest.

### Sampling and data collection

To assess priority woody species and carbon stock, we used social surveys (a participatory approach) [37] and inventory plots, respectively.

Prior to the start of the interviews, the goal of the study was first explained to village authorities and next to each of the informants in order to assure their participation. A pilot survey was conducted in each village (Cassou, Dao, Kou and Vrassan, see Fig 1), with 30 randomly selected respondents to determine the number of respondents to be interviewed. The sample size was determined afterwards for each village, following the normal approximation of the binomial distribution of Dagnelie [38]:

$$N=\frac{U_{1-\alpha /2}^{2}*p(1-p)}{d^{2}}$$ (1)

where *N* is the estimated sample size; *U* the value of the normal random variable (1.96 for α = 0.05), *p* the proportion of respondents who knew and used the preferred woody species; and *d* the predetermined margin of error for the survey (8%). The reason for using this sampling method is that it mostly targets households/informants of interest, and not the entire community.

The pilot survey revealed that 91% of respondents in Cassou and Kou, 90% in Dao and 89% in Vrassan used the preferred woody species and were knowledgeable about the species’ uses. Thus, for an error margin of 8%, 49, 50, 55 and 59 people were considered and randomly selected for the participatory group discussions in Cassou, Kou, Dao and Vrassan, respectively (Table 1).

**Table 1:** Number of localities in which surveys and questionnaires were administered to select priority woody species in the study area (Cassou, Dao, Kou and Vrassan) in Ziro province of Burkina Faso.

Village	Number of people interviewed		Total
	Men	Women	
**Cassou**	29	20	49
**Dao**	30	25	55
**Kou**	25	25	50
**Vrassan**	32	27	59
**Total**	116	97	213

A questionnaire was designed and administered to respondents. Information on priority woody species and their functions was collected through gendered (women and men) focus group discussions held in each village from July through August 2013, followed by individual interviews with key informants in each village.

The focus group discussions (FGD) were held to help enumerate different priority woody species and all the products and services provided by each species. Preferences reflect the usefulness of the species and may also reflect the abundance of the species in the village.

During the group discussions, information concerning products and services provided by woody species was obtained in four steps. First, the groups of men and women generated two lists of woody species they considered important, along with the products and services provided by each species. Secondly, each group discussed and reached a consensus on six priority tree functions, namely food, sale, medicine, fodder, energy, and craft. Thirdly, to qualify the functions of each species the groups assigned scores ranging from 0 to 3, with 0 corresponding to “not useful” and 3 to “very useful”. The importance of each species was estimated by calculating the sum of the six tree function scores, referred to hereafter as species importance value. The species importance values were then used to identify the priority species of the different social groups in each village. The importance of each function was determined by calculating the sum of its scores across all species. Lastly, the two gender groups came together to thoroughly review the priority species that each identified and reach a consensus about the list of priority woody species for the village. Thus, the list of priority species appears to reflect the local communities’ assessment and the species’ contributions to improving their livelihoods. Each FGD was carried out with 4–8 participants.

For the individual interviews, key informants (farmers, sellers and processors) were identified and interviewed to provide detailed information about preferences for products, their uses and the revenue earned. New priority species and functions quoted by the key informants were added to the list.

The vernacular names of woody species supplied by respondents in this study were crosschecked with previous studies: e.g. [39,40], as recommended by Nolan and Robbins [41]. The scientific names of the species and their authorities were validated using the website of West African Plants (http://www.westafricanplants.senckenberg.de/root/index.php) and Vascular Plants of Burkina Faso [39]. Interviews were conducted in two local languages (Mooré and Gourounsi) that are commonly spoken in the study areas. A field assistant who understood these languages acted as a translator and each interview session lasted between 1–2 hours. The present study was conducted in partnership with INERA and therefore validated as part of research programs by the scientific committee of “*Institut de l’Environnement et de la Recherche Agricole* (INERA)”. INERA has mandate and missions to generate scientific knowledge, technological innovations and decision support tools for improving agricultural sector in Burkina Faso (http://www.inera.bf/index.php/inera/missions). All procedures followed were in accordance with Helsinki Declaration of 1975, as revised in 2000. Informed consent was obtained from all participants included in the study.

The FGDs also helped to identify two sites (Cassou and Kou, see Fig 1) for biomass and carbon stocks estimation. Each sampling site covered 100 km^2^ (10 km x 10 km) where the Land Degradation and Surveillance Framework (LDSF) was applied [42]. A hierarchical stratified random sampling design was used in the sampling site consisting in 16 clusters of 100 ha each. Each cluster was composed of 10 plots of 0.1 ha established randomly. For this study, four subplots of 0.01 ha of 5.64 m radius as per a standard layout were randomly selected in each cluster for biomass sampling. Randomly selecting the plots eliminated the likelihood of convenience sampling and allowed to capture the representative mix of tree species in the study area.

Overall, 126 (44.06% of the total plots in the two sites) and 160 (55.94%) inventory plots were laid out in Cassou and Kou, respectively. In each subplot, individual trees were sampled for measurement using the T-square method, which is recommended for plant communities where individuals are randomly distributed [43–45]. Diameter at breast height (DBH) of all living trees (DBH≥5 cm) within the plot was measured at 1.3 m above the ground level, to the nearest 0.1 cm using a diameter tape. For trees forking below 1.3 m, the diameter of all ramifications was measured and DBH determined as the (quadratic mean diameter) square root of the sum of squares of individual stems. Tree height was measured from the base to the highest tip of the tree, to the nearest 0.1 m using a pole or a clinometer.

We estimated aboveground tree biomass (AGB) using the following generalized biomass estimation model developed for tropical forests [26]:

$$AGB={0.0673*(WD*{DBH}^{2}*H)}^{0.976}$$ (2)

Where AGB is the aboveground biomass per tree in kg per tree, *H* the height (m); DBH diameter at breast height; *WD* basic wood density (g. cm^-3^).

The *getWoodDensity* function from the BIOMASS package was used to assign a wood density value to each taxon using the global wood density database as a reference [46,47]. By default, *getWoodDensity* assigns to each taxon a species- or genus-level average if at least one wood density value in the same genus as the focal taxon is available in the reference database [48]. To upscale from the tree level to the site level (100 km^2^ or 10,000 ha), the predicted AGB was first calculated at the cluster scale (100 ha) and averaged based on the total number of plots. The AGB (Mg ha^-1^) was further upscaled to the study site level (10,000 ha) by applying the surface expansion factor (100). For carbon stock determination, the AGB (Mg ha^−1^) was converted by applying a carbon conversion factor of 0.5 [49,50]. Table 2 shows a descriptive summary of different variables measured.

**Table 2:** Descriptive statistic of structure and composition of trees in different vegetation types in Cassou and Kou.

Vegetation type	Forest	Woodland	Shrubland	Parkland agroforests
**Cassou**				
No. of plots	—	39	64	23
Area (ha)	—	0.39	0.64	0.23
No. of trees	—	407	566	95
Mean DBH (cm)		16.27±0.56	14.01±0.31	30.21±1.70
Range DBH (cm)		5.00–133.76	5.00–39.81	6.37–98.73
Mean tree height (m)		6.54±0.12	4.87±0.04	7.46±0.25
Range tree height (m)		3.10–17.00	1.80–6.90	3.20–16.10
Tree density (stems. ha^-1^)		1044	884	413
AGB (Mg. ha^-1^)		123.43±13.73	44.05±3.23	147.46±823.44
Range AGB (Mg. ha^-1^)		37.19–430.36	3.69–146.62	4.36–204.79
Carbon stocks (Mg. ha^-1^)		58.01±6.45	20.70±1.56	69.30±11.01
**Kou**				
No. of plots	53	32	22	53
Area (ha)	0.53	0.32	0.22	0.53
No. of trees	460	285	193	468
Mean DBH (cm)	18.93±0.48	19.97±0.68	11.59±0.34	23.56±0.59
Range DBH (cm)	5.09–50.96	5.20–55.41	5.09–27.07	5.20–59.87
Mean tree height (m)	8.12±0.15	7.37±0.15	5.09±0.08	7.17±0.10
Range tree height (m)	0.30–19.05	1.05–15.30	3.05–6.90	0.10–14.80
Tree density (stems. ha^-1^)	868	891	877	883
AGB (Mg. ha^-1^)	157.82±15.15	165.47±24.74	30.01±3.59	184.25±13.88
Range AGB (Mg. ha^-1^)	16.83–497.32	22.69–561.63	25.61–45.24	24.91–488.18
Carbon stocks (Mg. ha^-1^)	74.18±7.12	77.77±11.63	14.10±1.69	86.60±6.52

### Data processing and statistical analyses

Statistical analyses were done in the R statistical software package, version 3.3.2 [51]. Statistical assumptions were explored visually as proposed by Zuur et al. [52]. We first checked for normality among the different variables using the Shapiro–Wilk normality test.

To identify farmers’ preferences for trees and shrubs based on their functions in households, we next processed the data collected from the participatory group discussions to derive key variables, including the number of very useful or top-priority species, total species richness per gendered group, and tree function scores. The number of useful species reflects the diversity of products and services that a village depends on, and the number and proportion of species per function reflects the relative importance of the function in the village. Tree function scores and species importance values indicate the relative importance of the species for each function and across the six functions, respectively. To satisfy the assumptions of the analysis of variance, the number of very useful and the species richness per function in each village were transformed using square root. Because the number of very useful tree species, species richness and total score of tree function per village are count data, a generalized linear model (GLM) with Poisson error distribution was used to assess their variation with respect to village and gender. Because of the nature of the response variable (very useful tree species, species richness and total score of tree function), we performed separate GLM for each category (very useful tree species, species richness and total score of tree function). Village (Cassou, Dao, Kou and Vrassan) and gender (women vs men) were the predictors for the model. Using boxplots, we examined the effects of the villages and tree function on the (i) number of very useful species, (ii) species richness and (iii) tree function scores. Two Principal Component Analyses (PCA) based on tree function scores were used to assess the correlations between tree functions (medicine, food, sale, energy, fodder, and craft) and the priority woody species identified by local communities, and (ii) the relationships between tree functions and the gender groups across the four villages. The first two principal components were selected according to the latent root criterion [53]. The PCA were performed using the package FactoMineR in the R statistical software.

Differences between the carbon potential of the preferred, less preferred and not preferred species, of the two sites (Cassou and Kou) were analyzed using Kruskal–Wallis and post hoc pairwise Mann-Whitney tests using package ‘multcompView’ in R programming language 3.3.2 [51,54]. In all analyses, statistical significance was recognized at *p*<0.05.

## Results

### Floristic diversity and preferences for tree functions

**Fig 2:**
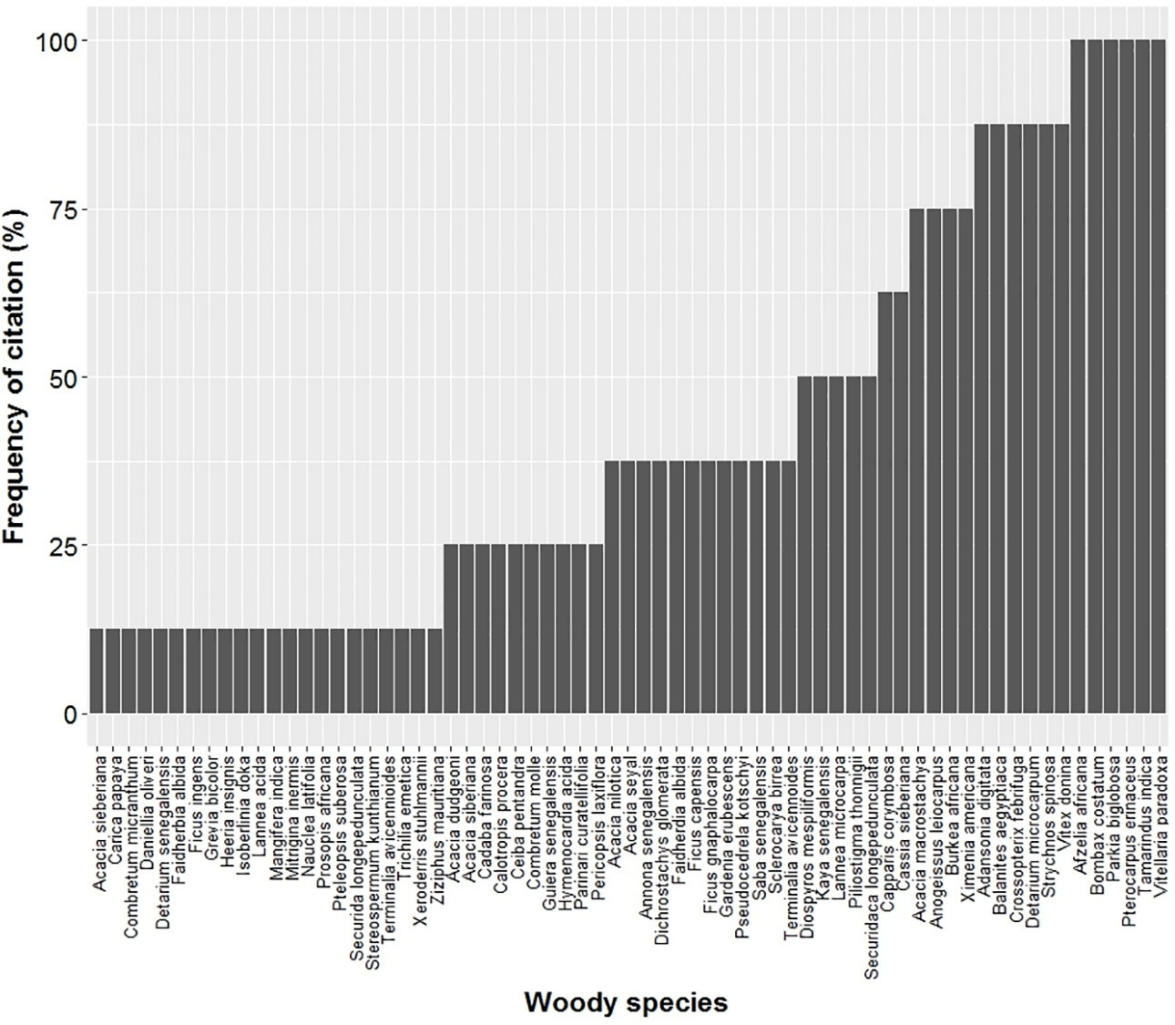
Frequency curve of species across the study villages (Cassou, Dao, Kou and Vrassan) in Ziro province of Burkina Faso.

In total, 79 woody species representing 65 genera and 26 families were listed by respondents in the four villages as important tree and shrub species (See S1 Table). The most represented families were Fabaceae-Mimosoideae (9 species), Fabaceae-Caesalpinioideae (9 species), followed respectively by Combretaceae (8 species), Anacardiaceae, Rubiaceae (6 species), Moraceae and Malvaceae (5 species). Among these important species, 18 were identified as top-priority species (Fig 2). *Afzelia africana* (100%: in all the 4 villages), *Bombax costatum* (100%), *Parkia biglobosa* (100%), *Pterocarpus erinaceus* (100%), *Tamarindus indica* (100%), *Vitellaria paradoxa* (100%) were mentioned by all respondents.

**Fig 3:**
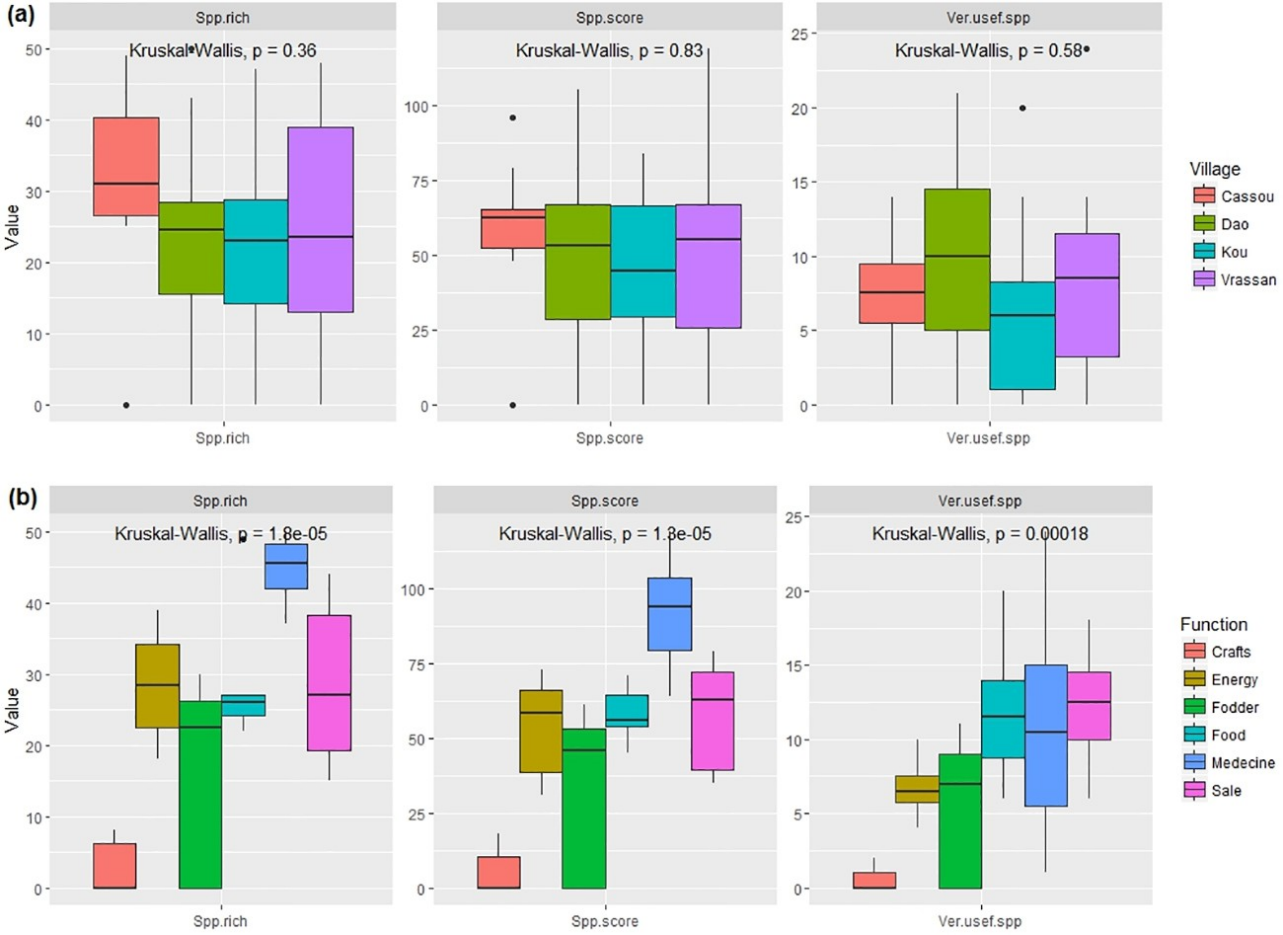
Abundance of very useful tree species, species richness and total score of tree function in Ziro province of Burkina Faso per (a) pilot village (Cassou, Dao, Kou and Vrassan) and (b) per tree function. Spp.rich = species richness; Spp.score = total score of tree function; Ver.usef.spp = very useful tree species.

Respondents listed “Medicine” (96%), “Food” (94%), “Sale” (87%), “Energy” (56%), “Fodder” (45%) and “Craft” (4%) as the main priority functions for tree and shrub species in the study area. There were no significant differences among villages in the number of very useful species (*p* = 0.58), species richness (*p* = 0.36) and tree function scores (*p* = 0.83) provided by the interviewees (Fig 3a). This means that the respondents in the four villages valued the species the same way giving function scores of similar magnitudes to the very useful species.

In contrast, the number of top priority woody species (*p* <0.001), total species richness (*p* < 0.00001) and total score (*p* < 0.001) differed significantly among the functions (Fig 3b). “Medicine” appeared as the most important function. In addition to being fulfilled by a highest number of top-priority species (11.3±2.9), this function displayed the highest total species richness (44.6±1.7) and highest functional score (91.0±6.8) (Fig 3b). In decreasing order, “Food”, “Sale”, “Energy”, “Fodder” and “Craft” followed “Medicine” in terms of importance.

In the PCA-biplot, the first two axes accounted for 67.6% of observed variation (Table 3) and thus were used to describe the relationships between tree functions and woody species. The first axis was positively and significantly correlated with “Sale”, “Food” and “Energy”, while the second axis was positively and significantly correlated with “Fodder” and negatively correlated with “Medicine” (Table 3).

**Table 3:** Description of the PCA dimensions by their correlation coefficients with the tree functions listed by the discussion group participants in the four villages (Cassou, Dao, Kou and Vrassan) in Ziro province of Burkina Faso.

Variable (Tree function)	Axis 1 (38.7%)		Axis 2 (28.9%)	
	Correlation	*p*	Correlation	*p*
**Energy**	0.56	0.021	—	—
**Fodder**	—	—	0.84	<0.001
**Food**	0.75	<0.001	0.43	0.022
**Medicine**	0.46	0.014	-0.65	<0.001
**Sale**	0.92	0.041	—	—

**Fig 4:**
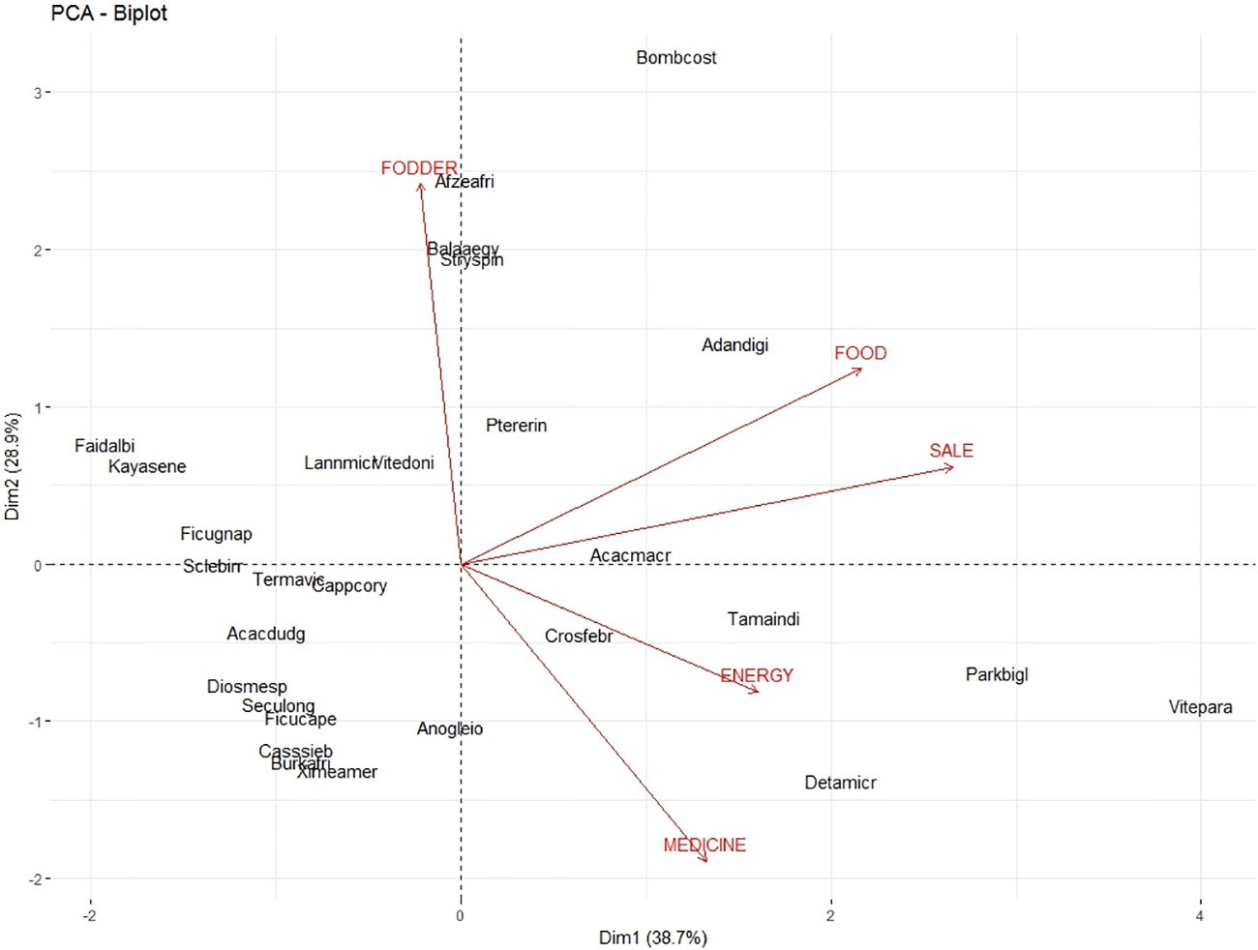
Biplot of the Principal Component Analysis assessing the relationship between woody species and attributes assigned by the four villages (Cassou, Dao, Kou and Vrassan) in Ziro province of Burkina Faso. Afzeafri: *Afzelia africana*, Balaaegy: *Balanites aegyptiaca*, Stryspin: *Strychnos spinosa*, Faidalbi: *Faidherbia albida*, Kayasene: *Khaya senegalensis*, Ficugnap: *Ficus gnaphalocarpa*, Sclebirr: *Sclerocarya birrea*, Termavic: *Terminalia avicennioides*, Cappcory: *Capparis corymbosa*, Acadudg: *Acacia dudgeonii*, Diosmesp: *Diospyros mespiliformis*, Seculong: *Securidaca longipedunculata*, Ficucape: *Ficus capensis*, Casssieb: *Cassia sieberiana*, Burkafri: *Burkea africana*, Ximeamer: *Ximenia americana*, Anogleio: *Anogeisus leiocarpa*, Crosfebr: *Crossopteryx febrifuga*, Lannmicr: *Lannea microcarpa*, Vitedoni: *Vitex doniana*, Ptererin: *Pterocarpus erinaceus*, Acamacr: *Acacia macrostachya*, Adandigi: *Adansonia digitata*, Tamaindi: *Tamarindus indica*, Detamicr: *Detarium microcarpum*, Parkbigl: *Parkia biglobosa*, Vitepara: *Vitellaria paradoxa*.

Based on the correlations (Table 3), the first axis primarily reflects food, while the second axis primarily reflects fodder and medicine. Furthermore, it revealed relationships between “Food” and “Sale”, and between “Energy” and “Medicine” with “Fodder” representing the third isolated group (Fig 4). Axis 1 was driven by the association of “Sale”, “Food”, “Energy”, and “Medicine” with species such as *V*. *paradoxa*, *P*. *biglobosa*, *Detarium microcarpum*, *Acacia macrostachya*, *Adansonia digitata* and *T*. *indica*. On the other hand, *B*. *costatum*, *A*. *africana*, *Balanites aegyptiaca* and *Strychnos spinosa* tended to cluster with “Fodder”, the primary contributing factor in the construction of Axis 2.

The top species, with the highest ranking in each village and for each gendered group, are presented in Table 4. The top 28 species listed by respondents were present in all villages, but listing the species and species preferences varied according to gender and from one village to another. Men and women together in Vrassan named the most species, with 20 out of 28 listed as preferred. Species that were less and not preferred are not presented here, but are considered in the section related to carbon stocks.

**Table 4:** Scoring of the preferred tree species according to the gender in each village (Cassou, Dao, Kou and Vrassan) in Ziro province of Burkina Faso.

Species	Cassou		Dao		Kou		Vrassan	
	Men	Women	Men	Women	Men	Women	Men	Women
*Acacia dudgeoni*							3	
*Acacia macrostachya*			1	3				4
*Acacia sieberiana*	7						7	
*Adansonia digitata*	5	4	8		6	3		7
*Afzelia africana*	7	1			1		3	
*Anogeissus leiocarpa*			10	7				7
*Balanites aegyptiaca*		7	6	7				
*Bombax costatum*	2	4	1	3	4	3		
*Capparis corymbosa*						8		7
*Ceiba pentandra*		10						
*Crossopteryx febrifuga*				10			3	
*Detarium microcarpum*	5	4	1	2	6	1		1
*Ficus capensis*							1	
*Ficus gnaphalocarpa*							3	
*Ficus ingens*							7	
*Isoberlinia doka*							7	
*Kaya senegalensis*	7				9			
*Lannea microcarpa*							2	
*Mangifera indica*						8		
*Parkia biglobosa*	2	1	1	3	4	3		1
*Pericopsis laxiflora*							7	
*Pterocarpus erinaceus*	2			3	6	8		6
*Saba senegalensis*						3		
*Strychnos spinosa*	7	7	6	7	1			
*Tamarindus indica*			8			3		4
*Terminalia avicennioides*								7
*Vitellaria paradoxa*	1	1	1	1	3	2		1
*Vitex doniana*		7			9			

*V*. *paradoxa* was the favorite species across the four villages, closely followed by *P*. *biglobosa*, *B*. *costatum*, *D*. *microcarpum*, *A*. *digitata* and *S*. *spinosa*. These species were scored in the top ten in three villages. Four species (*T*. *indica*, *B*. *costatum*, *A*. *africana* and *S*. *spinosa*) were cited in three villages, and eight species (*Capparis corymbosa*, *Vitex doniana*, *Khaya senegalensis*, *Crossopteryx febrifuga*, *Balanites aegyptiaca*, *Anogeissus leiocarpa*, *Acacia sieberiana* and *Acacia macrostachya*) were preferred in two villages. Eleven species (*Terminalia avicennioides*, *Saba senegalensis*, *Pericopsis laxiflora*, *Mangifera indica*, *Lannea microcarpa*, *Isoberlinia doka*, *Ficus ingens*, *Ficus gnaphalocarpa*, *Ficus capensis*, *Ceiba pentandra* and *Acacia dudgeonii*) were listed only in one village. It is noteworthy that only one of the 28 species (*Mangifera indica*) is exotic to Burkina Faso. The general interest in the 28 species revealed by the discussion groups augurs well for local tree species domestication initiatives.

### Gender differentiated species preferences

The results of the GLM showed that the number of very useful species, species richness and tree function varied significantly with gender (Table 5). In addition, gender was involved in significant interactions with village (Table 5). The number of very useful species did not differ significantly among the four villages (Cassou, Dao, Kou and Vrassan), but species richness and tree function scores did differ significantly among villages (Table 5).

**Table 5:** Results of the generalized linear models showing the relationship between gender and village, with useful tree species, species richness and tree function score in Ziro province of Burkina Faso.

Predictors	Very useful species			Species richness			Tree function score		
	df	dev.	*p*-value	df	dev.	*p*-value	df	dev.	*p*-value
Village	3	0.17	0.68	3	12.57	<0.001	3	16.8	<0.001
Gender	1	9.17	0.027	1	19.5	<0.001	1	14.7	0.002
Village x Gender	3	12.4	<0.01	3	13.73	<0.001	3	30.76	<0.001

df: degree of freedom, dev.: deviance, *p*- value: probability value computed from a Chi-square distribution

**Fig 5:**
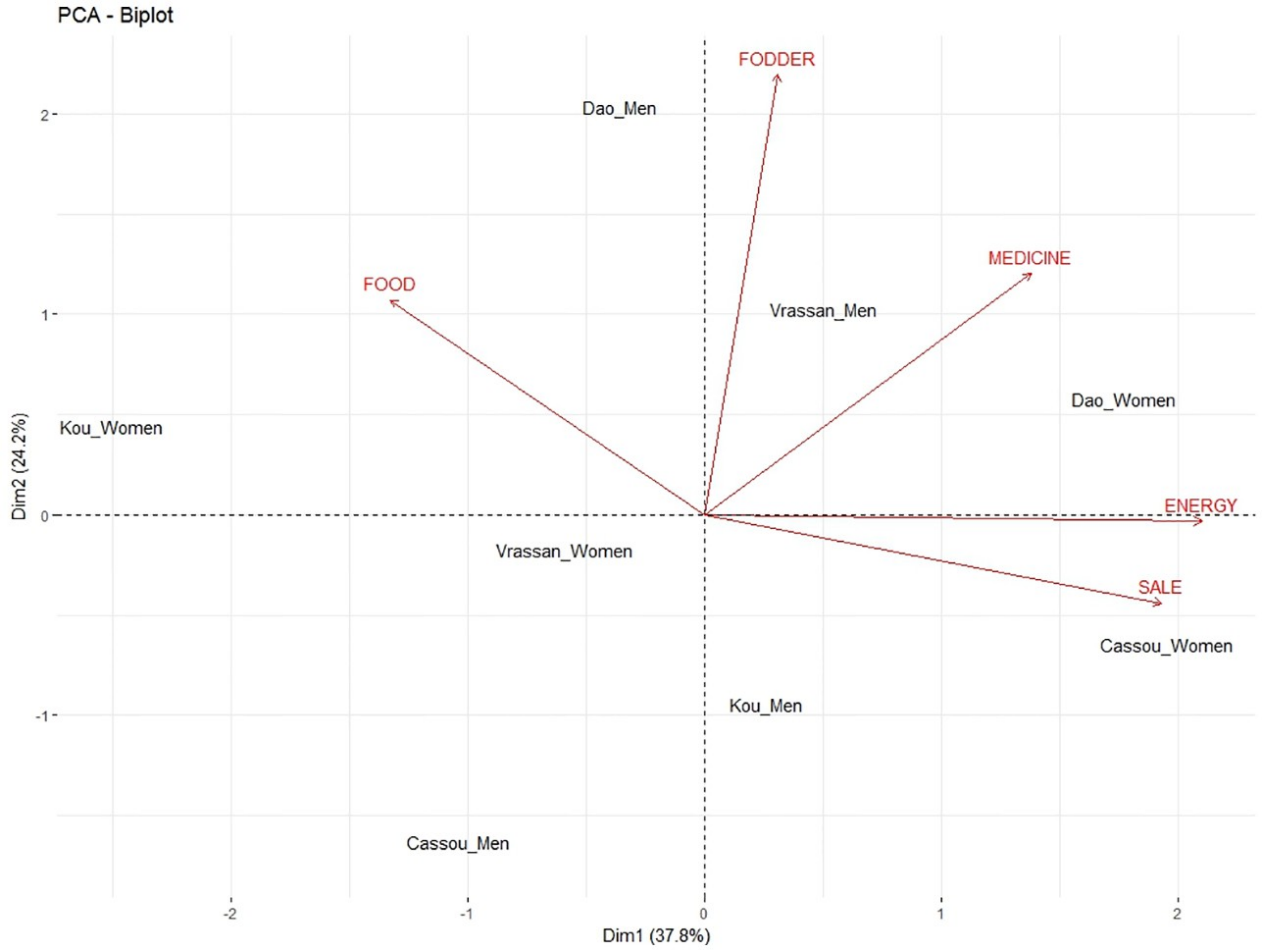
Biplot of the Principal Component Analysis assessing the relationship between gendered groups and woody vegetation attributes in the studied landscape of the four villages (Cassou, Dao, Kou and Vrassan) in Ziro province of Burkina Faso.

The PCA biplot with tree functions and gender is shown in Fig 5. The first two axes of the PCA biplot accounted for a total variance of 67.6%. Woody vegetation attributes such as “Energy” and “Sale” were significantly correlated to Axis 1 of the biplot, indicating that women are strongly related to the collection of wood for fuelwood used for their own needs and for sale of the surplus to generate income (Table 6; Fig 5). The second axis correlated positively and significantly with “Fodder,” showing men’s preferences, attributable to their responsibilities for feeding household livestock.

**Table 6:** Description of the PCA dimensions by their correlation coefficients with the tree functions listed by the respondents of the four villages (Cassou, Dao, Kou and Vrassan) in Ziro province of Burkina Faso and the value of measured variables (abundance of very useful tree species, tree species richness and total score of tree function).

Variable (Tree functions)	Axis 1 (38.7%)		Axis 2 (28.9%)	
	Correlation	*p*	Correlation	*p*
**Energy**	0.84	0.010	—	—
**Fodder**	—	—	0.87	0.0045
**Food**	—	—	—	—
**Medicine**	—	—	—	—
**Sale**	0.77	0.025	—	—

### Carbon stock potential for priority woody species

The DBH and height of 1,068 and 1,406 individuals of the preferred woody species were measured in Cassou and Kou, respectively (Table 1). In the four land use and land cover categories (LULCc) considered in this study, *V*. *paradoxa*, often associated with *D*. *microcarpum*, was the species most encountered (Table 7).

**Table 7:** Top five dominant woody species per land use and land cover category in Cassou and Kou in Ziro province of Burkina Faso (values in parentheses are the number of individuals).

Site	Parkland agroforests	Shrubland	Woodland	Forest
Cassou	*Vitellaria paradoxa (52)*	*Vitellaria paradoxa (71)*	*Vitellaria paradoxa (52)*	*—*
	*Parkia biglobosa (09)*	*Detarium microcarpum (64)*	*Detarium microcarpum (44)*	*—*
	*Lannea acida (04)*	*Combretum molle (37)*	*Crossopteryx febrifuga (35)*	*—*
	*Detarium microcarpum (02)*	*Lannea acida (31)*	*Piliostigma thonningii (31)*	*—*
	*Lannea microcarpa (02)*	*Crossopteryx febrifuga (30)*	*Terminalia laxiflora (27)*	*—*
Kou	*Vitellaria paradoxa (272)*	*Vitellaria paradoxa (46)*	*Vitellaria paradoxa (67)*	*Anogeisus leiocarpa (114)*
	*Bombax costatum (22)*	*Piliostigma thonningii (14)*	*Detarium microcarpum (60)*	*Detarium microcarpum (65)*
	*Sterculia setigera (17)*	*Detarium microcarpum (11)*	*Anogeisus leiocarpa (24)*	*Vitellaria paradoxa (42)*
	*Terminalia aviceniodes (15)*	*Acacia macrostachya (10)*	*Combretum fragrans (18)*	*Acacia macrostachya (27)*
	*Crossopteryx febrifuga (12)*	*Terminalia aviceniodes (10)*	*Burkea africana (15)*	*Combretum fragrans (26)*

The estimate average aboveground carbon stocks (AGC) varied from 14.1 ±1.7 to 86.6 ± 6.5 MgC ha^-1^ (±indicates standard error throughout) across the LULCc of Cassou and Kou (see Table 2). The lowest value of AGC was in shrubland in Kou (14.1±1.7 MgC ha^-1^) and the highest value in parkland agroforests in Kou (86.6 ± 6.5 MgC ha^-1^). Using a pairwise comparison (Wilcoxon test), there was a significant difference between the amount of AGC stored in shrubland (*p* <0.0001) and woodland of the two sites (*p* <0.0001). However, there was no significant difference in the amount of AGC stored in parkland agroforests (p = 0.34). Forests were encountered only in Kou and they stored 74.2 ±7.1 MgC ha^-1^ of AGC. As the cultivated land is estimated to be 20.4% and 68.9% in Cassou and Kou, respectively, their respective parkland agroforests could potentially store 1695.8 Mg C ha^-1^ and 4882.6 Mg C ha^-1^ in aboveground biomass.

**Fig 6:**
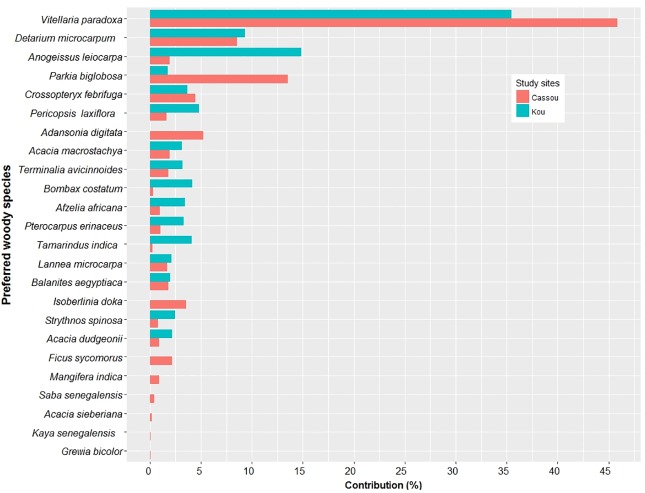
Contribution of preferred woody species to the pool of carbon stock in Cassou and Kou in Ziro Province of Burkina Faso.

Among the preferred woody species studied, *V*. *paradoxa* stored the highest quantity of carbon in the two sampling sites (1180.0 ±208.5 to 2088.8±522.2 kg C ha^-1^) while the lowest stocks were found in Cassou with *Tamarindus indica* (0.7±0.7 kg C ha^-1^) and Kou with *Grewia bicolor* (4.7±1.2 kg C ha^-1^) (S2 Table). In Cassou, the highest carbon stocks were stored by *V*. *paradoxa*, *A*. *digitata*, *P*. *biglobosa*, *D*. *microcarpum*, *C*. *febrifuga*, *Piliostigma thonningii*, *Ficus sycomorus*, *Isoberlinia doka and Terminalia laxiflora* while *V*. *paradoxa*, *T*. *indica*, *A*. *leiocarpa*, *B*. *costatum*, *P*. *erinaceus*, *A*. *africana*, *C*. *febrifuga* and *D*. *microcarpum* displayed the greatest values in Kou (S2 Table). When assessing the contribution of all species (preferred, less preferred and not preferred) to the total pool of aboveground carbon, we found that in Kou three of the 57 species contributed 60% while in Cassou five of the 72 species contributed 60%. *V*. *paradoxa* stored the largest percentage of carbon in Cassou (34.3%) and Kou (41.2%), followed by *P*. *biglobosa* (10.1%) and *D*. *microcarpum* (6.4%) in Cassou, *A*. *leiocarpa* (12.9%) and *D*. *microcarpum* (6.5%) in Kou (Fig 6).

The potential of carbon storage by the preferred species is significantly higher compared to that of less and not preferred species listed by the respondents (Table 8), indicating that preferred species could positively influence biocarbon projects in the study areas. The lowest amounts of carbon stocks were recorded for *Holarrhena floribunda* (3.0 ± 0.7 Kg C ha^-1^) and *Hymenocardia acida (*0.1 ± 0.2 Kg C ha^-1^) which are not preferred species by farmers in Kou and Cassou, respectively (S2 Table). The Wilcoxon test statistics showed significant differences in the carbon stored by the preferred woody species in Cassou and Kou (*p* <0.0001). The potential of AGC stored by the preferred woody species (see S2 Table for number of preferred species) was estimated at 587.9 Mg C (95% CI: [456.8; 719.1 Mg C]) in Kou and 256.8Mg C (95% CI: [67.6; 324.4 Mg C]) in Cassou.

**Table 8:** Mean values of carbon potential of the preferred, less preferred and not preferred species by the respondents across Cassou and Kou in Ziro province in Burkina Faso.

Sites	Mean carbon (Mg C ha^-1^)		
	Preferred species	Less preferred species	Not preferred species
Cassou	256.80 ±31.71a	107.32 ± 25.10b	13.53 ± 3.34c
Kou	587.94±61.55a	152.38±14.86b	21.72±10.96c

Different letters indicate significant differences (pairwise Mann–Whitney test, p < 0.05)

## Discussion

### Functions of smallholders preferred woody species

The overall species richness listed by respondents in this study accounts for 29.3% of all native woody species in Burkina Faso, reported in a previous study as 376 species (including 96 exotic species), 214 genera and 55 families [39]. More than 98% of those listed by the respondents are indigenous species that farmers protect or nurture in their farms. The high species richness underscores the importance of these species to the rural communities who rely on them to sustain their livelihoods. This finding confirms information from other studies about the critical role of traditional agroforestry practices in supporting biodiversity through *in situ* conservation of tree species [55,56]. This number of woody species cited by respondents also is indicative of the extent to which local people serve as a repository of knowledge on their local vegetation. Previous studies reported that local knowledge can provide important information on plant species richness, diversity, abundance and rarity, necessary for understanding not only local vegetation dynamics but also fundamental management strategies for sustainable use and conservation of natural vegetation [57,58].

Among the top 28 woody species that respondents listed in this study, 18 were identified as top-priority because of their role in providing products and services to sustain livelihoods (“Food”, “Energy”, “Sale”, “Fodder”) and health status (“Medicine”) of smallholders. Local communities prefer most species identified as priority owing to their multipurpose functions providing several products and services, including many income sources. This confirms the statement of Faye et al. [32] about the yearly contribution of agroforestry products (as high as 650 USD) in sustaining household livelihoods in the Sahel. For instance, local communities highly appreciate the foods and condiments provided by *V*. *paradoxa*, *P*. *biglobosa*, *T*. *indica* and *A*. *digitata*. Previous studies have reached similar conclusions about the socioeconomic importance of these species in West Africa [59–62]. Women greatly value *V*. *paradoxa* nuts for their high oil content, and in all four villages they listed *A*. *digitata* as top-priority because the leaves are an important sauce ingredient consumed throughout the year, also reported in Faye et al. [37] for smallholders in Mali, Burkina Faso, Niger and Senegal. The critical role *A*. *africana*, *P*. *erinaceus*, and *B*. *aegyptiaca* play in providing fodder elicited men’s top-priority ranking. This finding agrees with that of Naah et al. [63] who reported that men are a repository of local knowledge on fodder. These trees also supply farmers with fuelwood, as well as several other services [64–66].

Registering “Medicine” (96%) and “Food” (94%) as top-priority functions indicates smallholders’ knowledge of trees for nutritional and health properties. Smallholders’ reliance on more accessible and affordable herbal medicines for health care illustrates the major challenges that health care presents in Burkina Faso [67]. These categories were followed by “Sale” (87%), “Energy” (56%), and “Fodder” (45%), consistent with the prime importance of these functions for household income generation, fuel and livestock feed [32,37]. The study revealed a gender-oriented preference for tree species and functions in all study villages. Overall, while men focused on provisioning services to feed their livestock, women focused on energy (fuel wood) and income.

The positive relationships among most woody species’ functions indicate their multiple uses for smallholders in the West African Sahel [37]. Some fruits, fats, leafy vegetables, nuts and condiments may be used as food, to cure certain diseases [37], and sold for cash.

### Carbon stock potential of preferred woody species in rural communities

In this study, *V*. *paradoxa*, *P*. *biglobosa*, *D*. *microcarpum*, *A*. *leiocarpa*, *C*. *febrifuga* and *B*. *costatum* stored more carbon than the other preferred woody species in Kou and Cassou. This could be explained by the fact that these species were more abundant and their dbh was larger than the dbh of the other preferred woody species. Apart from *A*. *leiocarpa* and *B*. *costatum* which are commonly found in natural vegetation, *V*. *paradoxa*, *P*. *biglobosa*, and *D*. *microcarpum* are encountered in parklands. *V*. *paradoxa* and *P*. *biglobosa* are two of the dominant tree species of parklands in Burkina Faso [68]. They have great socioeconomic importance for rural communities as well as ecological functions, providing a range of ecosystem services, including shade and habitat for biodiversity (other plant and animal species), and carbon storage [5,37]. The capacity of these two species to supply goods and services to the local people confers upon them both systematic protection against anthropogenic interventions, such as excessive harvesting and pruning, and enhanced restoration through farmer-managed natural regeneration (FMNR).

Carbon stocks varied greatly among woody species owing to the different population densities per cluster and their morphological differences. In addition, there were large differences in carbon stocks between the two sites for species like *V*. *paradoxa*, *A*. *leiocarpa*, *B*. *costatum*, *Pericopsis laxiflora*, and *P*. *biglobosa*, possibly due to factors that were not assessed in this study, such as microclimate, tree size (due to age differences and /or pruning), abundance of the species or genetic differences in tree growth [69]. The carbon stock obtained in Kou for *A*. *leiocarpa*, for example, is far lower than the 907.9 kg C ha^-1^ reported by Eneji et al.[70] in a Forest Reserve of Benue State, Nigeria, and that stored by *P*. *biglobosa* in the same forest (301.6 kg C ha^-1^) is slightly lower that that measured in Cassou (372.3±111.0 kg C ha^-1^). The estimated total carbon stored in studied parklands in Cassou (1695.8 Mg C ha^-1^) and Kou (4882.6 Mg C ha^-1^) is lower than the amount estimated for mature parklands of the West Afican Sahel (1,284 Tg: [18]).

Three reasons could explain the lower values obtained in our study. First, measuring only trees with heights ≥3 m reduced the number of inviduals included in the sample.. Second, the use of average genus and family wood density in the absence of species wood density could have been a source of error as wood density of species within the same genus can differ greatly [20]. Third, due to higher land-use pressure in the study sites as compared to the sites of previous studies (e.g Forest Reserve of Benue State), most of the trees are thinned. The aggregated impact of these sources of errors could have resulted in much lower carbon stock values for each species and for the overall landscapes. The significantly higher stock of carbon in trees in Kou relative to Cassou is likely due to Kou’s high density of *V*. *paradoxa* and *A*. *leiocarpa*. Cassou has much better market access, intensifying anthropogenic pressure on woody species there as opposed to Kou. Furthermore, among the land use categories considered in this study, the highest overall mean AGC stock was recorded for parkland agroforests. This can be attributed to the fact that parkland agroforests in the two sites were mainly composed of large *V*. *paradoxa* trees, and *V*. *parado*xa was the most abundant species amongst all the woody species measured in both sites. The AGC stocks recorded in woodland and shrubland were higher in Cassou compared to Kou. This difference could be explained by the management system of the two sites and the variability of dendrometric parameters in the individuals of the same species for different geographical locations and ages [71].

### Implications for biocarbon projects

Based on this and other studies, we recommend the following for biocarbon projects in Burkina Faso: (i) use the most preferred tree and shrub species for FMNR on farmed lands, since these are the species that farmers are most likely to maintain in their landscapes; (ii) plant the preferred species in degraded parklands using selected germplasm to increase their production over time. Because the climate is becoming hotter and drier and with more variability in rainfall, it is recommended to use germplasm (i.e., seeds, rooted cuttings, etc.) originating from drier locations within the same general region [69]; (iii) train smallholders in tree domestication techniques to promote the species with high carbon sequestration potential; and (iv) promote afforestation and reforestation within the degraded portions of Cassou Protected Forest.

## Conclusion

This study highlighted the importance of woody species in local people’s strategies to sustain their livelihoods, which may be instrumental to climate change adaptation. Local people in the four villages knew of and used woody species (trees and shrubs) for several functions, but knowledge of woody species varied according to species’ attributes and smallholders’ gender. Preferred woody species in Cassou and Kou store more carbon in their biomass compared to less preferred and unpreferred species listed by respondents. Hence, for biocarbon initiatives, it is recommended to use preferred species such as *V*. *paradoxa*, *A*. *leiocarpa*, *P*. *biglobosa*, and *D*. *microcarpum* owing to their appreciable carbon storage potential. Kou had greater potential for carbon storage than Cassou. The challenge now is to determine a cost-effective way to involve local men and women together with technical teams from research institutes and universities in participatory tree domestication and smart land use programs which rely on innovative smallholder-based extension approaches.

The carbon stocks estimated in the current study are subject to the limitations associated with use of a general allometric equation, wood density values from literature, and a wood carbon concentration estimate of 50%. The results should therefore be considered preliminary until confirmed by future studies that use species-specific allometric equations and estimates for wood density and carbon concentration based on studies across a range of land use types, soil types and mean annual rainfall. The variation in carbon stock values across the land use types in Cassou and Kou is corroborated by the influence of structural variables (DBH, and tree height). For instance, the highest amount of carbon stock was recorded in the two sites where the largest trees were measured. This result can be used to design management and conservation plans. For instance, the finding that the largest tree species greatly influenced the carbon stock suggests that conservation of trees with larger size (especially those that farmers are more likely to conserve in their landscapes such as trees for food and fodder) could be a strategy to increase carbon storage. As the largest trees will die in time, an appropriate management strategy would be needed to protect smaller trees (regeneration) in order to maintain a balanced vegetation structure over time.

## Supporting information

S1 Dataset Data for Figs 2, 3, 4, 5 and 6. (RAR)

S2 Dataset Dendrometric parameters used to compute aboveground biomass and carbon in Cassou and Kou. (XLSX)

S1 Table List of plant species recorded in the four villages (Cassou, Dao, Kou and Vrassan) in Ziro province of Burkina Faso. (DOCX)

S2 Table Estimated aboveground carbon stock (Average ± SE) of preferred species, less preferred and not preferred species at cluster level in Cassou and Kou in Ziro province of Burkina Faso. (DOCX)
