# Supplementary material for: Farmers’ preferred tree species and their potential carbon stocks in southern Burkina Faso: Implications for biocarbon initiatives
Source: PLoS One. 2018 Dec 18;13(12):e0199488. doi: 10.1371/journal.pone.0199488 (PMC6298700; doi:10.1371/journal.pone.0199488)
Supplement: S1 Table — (DOCX) [file pone.0199488.s003.docx]

S1 Table. List of plant species recorded in the four villages (Cassou, Dao, Kou and Vrassan) in Ziro province of Burkina Faso

| Species | Family |
| --- | --- |
| *Afzelia africana* Sm. ex Pers. | Fabaceae-Caesalpinioideae |
| *Acacia dudgeonii* Craib ex Holland | Fabaceae-Mimosoideae |
| *Acacia macrostachya* Rchb. ex DC. | Fabaceae-Mimosoideae |
| *Acacia nilotica* (L.) Willd. ex Delile | Fabaceae-Mimosoideae |
| *Acacia seyal* Delile | Fabaceae-Mimosoideae |
| *Acacia sieberiana* DC. | Fabaceae-Mimosoideae |
| *Adansonia digitata* L. | Malvaceae |
| *Annona senegalensis* Pers. | Annonaceae |
| *Anogeissus leiocarpa* (DC.) Guill. & Perr. | Combretaceae |
| *Balanites aegyptiaca* (L.) Delile | Zygophyllaceae |
| *Bombax costatum* Pellegr. & Vuill. | Malvaceae |
| *Burkea africana* Hook. | Fabaceae-Caesalpinioideae |
| *Cadaba farinosa* Forssk. | Capparaceae |
| *Calotropis procera* (Aiton) R.Br. | Apocynaceae |
| *Capparis corymbosa* Lam. | Capparaceae |
| *Carica papaya* L. [cult.] | Caricaceae |
| *Cassia sieberiana* DC. | Fabaceae-Caesalpinioideae |
| *Ceiba pentandra* (L.) Gaertn. | Malvaceae |
| *Combretum micranthum* G.Don | Combretaceae |
| *Combretum molle* R.Br. ex G.Don | Combretaceae |
| *Crossopteryx febrifuga* (Afzel. ex G.Don) Benth. | Rubiaceae |
| *Daniellia oliveri* (Rolfe) Hutch. & Dalziel | Fabaceae-Caesalpinioideae |
| *Detarium microcarpum* Guill. & Perr. | Fabaceae-Caesalpinioideae |
| *Detarium senegalense* J.F.Gmel. | Fabaceae-Caesalpinioideae |
| *Dichrostachys cinerea* (L.) Wight & Arn. | Fabaceae-Mimosoideae |
| *Diospyros mespiliformis* Hochst. ex A.DC. | Ebenaceae |
| *Faidherbia albida* (Delile) A.Chev. | Fabaceae-Mimosoideae |
| *Ficus capensis* Thunb. | Moraceae |
| *Ficus gnaphalocarpa* (Miq.) A.Rich. | Moraceae |
| *Ficus ingens* (Miq.) Miq. | Moraceae |
| *Gardenia erubescens* Stapf & Hutch. | Rubiaceae |
| *Grewia bicolor* Juss. | Malvaceae |
| *Guiera senegalensis* J.F.Gmel. | Combretaceae |
| *Heeria insignis* (Delile) Kuntze | Anacardiaceae |
| *Hymenocardia acida* Tul. | Phyllanthaceae |
| *Isoberlinia doka* Craib & Stapf | Fabaceae-Caesalpinioideae |
| *Khaya senegalensis* (Desr.) A.Juss. | Meliaceae |
| *Lannea acida* A.Rich. | Anacardiaceae |
| *Lannea microcarpa* Engl. & K.Krause | Anacardiaceae |
| *Mangifera indica* L. [cult.] | Anacardiaceae |
| *Mitragyna inermis* (Willd.) Kuntze | Rubiaceae |
| *Nauclea latifolia* Sm. | Rubiaceae |
| *Parinari curatellifolia* Planch. ex Benth. | Chrysobalanaceae |
| *Parkia biglobosa* (Jacq.) R.Br. ex G.Don | Fabaceae-Mimosoideae |
| *Pericopsis laxiflora* (Benth.) Meeuwen | Fabaceae-Faboideae |
| *Piliostigma thonningii* (Schumach.) Milne-Redh. | Fabaceae-Caesalpinioideae |
| *Prosopis africana* (Guill. & Perr.) Taub. | Fabaceae-Mimosoideae |
| *Pseudocedrela kotschyi* (Schweinf.) Harms | Meliaceae |
| *Pteleopsis suberosa* Engl. & Diels | Combretaceae |
| *Pterocarpus erinaceus* Poir. | Fabaceae-Faboideae |
| *Saba senegalensis* (A.DC.) Pichon | Apocynaceae |
| *Sclerocarya birrea* (A.Rich.) Hochst. | Anacardiaceae |
| *Securidaca longipedunculata* Fresen. | Polygalaceae |
| *Stereospermum kunthianum* Cham. | Bignoniaceae |
| *Strychnos spinosa* Lam. | Linderniaceae-Loganiaceae |
| *Tamarindus indica* L. | Fabaceae-Caesalpinioideae |
| *Terminalia avicennioides* Guill. & Perr. | Combretaceae |
| *Trichilia emetica* Vahl | Meliaceae |
| *Vitellaria paradoxa* C.F.Gaertn. | Sapotaceae |
| *Vitex doniana* Sweet | Lamiaceae |
| *Xeroderris stuhlmannii* (Taub.) Mendonça & E.C.Sousa | Fabaceae-Faboideae |
| *Ziziphus mauritiana* Lam. | Rhamnaceae |
