# Supplementary material for: Farmers’ preferred tree species and their potential carbon stocks in southern Burkina Faso: Implications for biocarbon initiatives
Source: PLoS One. 2018 Dec 18;13(12):e0199488. doi: 10.1371/journal.pone.0199488 (PMC6298700; doi:10.1371/journal.pone.0199488)
Supplement: S2 Table — (DOCX) [file pone.0199488.s004.docx]

S2 Table. Estimated aboveground carbon stock (Average ± SE) of preferred species, less preferred species, and not preferred species at cluster level in Cassou and Kou in Ziro province of Burkina Faso

| **Species** | **Cassou** | **Kou** |  |
| --- | --- | --- | --- |
| **Preferred species** | **Carbon (kg C ha^-1^)** | **Carbon (kg C ha^-1^)** |  |
| *Acacia dudgeonii* | 24.10±8.03 | 127.64±31.91 |  |
| *Acacia macrostachya* | 50.31±14.91 | 185.86±46.47 |  |
| *Acacia sieberiana* | 5.33±2.49 |  |  |
| *Adansonia digitata* | 134.66±134.48 |  |  |
| *Afzelia africana* | 24.74±13.45 | 201.79±50.45 |  |
| *Anogeissus leiocarpa* | 49.64±20.54 | 873.37±218.34 |  |
| *Balanites aegyptiaca* | 46.33±23.75 | 115.83±28.96 |  |
| *Bombax costatum* | 8.71±3.70 | 244.75±61.19 |  |
| *Crossopteryx febrifuga* | 114.49±29.04 | 215.60±53.90 |  |
| *Detarium microcarpum* | 219.73±51.11 | 549.82±137.46 |  |
| *Isoberlinia doka* | 91.13±50.08 |  |  |
| *Kaya senegalensis* | 1.15±1.15 |  |  |
| *Lannea microcarpa* | 43.78±30.08 | 123.32±30.83 |  |
| *Mangifera indica* | 22.82±22.82 |  |  |
| *Parkia biglobosa* | 348.36±130.51 | 102.90±25.72 |  |
| *Pericopsis laxiflora* | 41.71±17.23 | 282.72±70.68 |  |
| *Pterocarpus erinaceus* | 27.27±14.01 | 194.52±48.63 |  |
| *Saba senegalensis* | 10.42±10.22 |  |  |
| *Strythnos spinosa* | 20.20±10.66 | 144.64±36.16 |  |
| *Tamarindus indica* | 0.67±0.67 | 240.25±60.06 |  |
| *Terminalia avicinnoides* | 46.96±24.50 | 189.01±47.25 |  |
| *Vitellaria paradoxa* | 1180.04±208.51 | 2088.83±522.21 |  |
| *Ficus sycomorus* | 55.42±31.98 |  |  |
| *Grewia bicolor* |  | 4.74±1.19 |  |
| **Less preferred species** | | | |
| *Annona senegalensis* | 0.31±0.31 | 5.24±1.31 |  |
| *Combretum collinum* | 6.93±6.93 |  |  |
| *Combretum glutinosum* | 38.84±11.23 | 128.06±32.02 |  |
| *Combretum molle* | 55.12±11.98 | 241.56±60.39 |  |
| *Combretum nigricans* | 33.82±17.17 | 10.37±2.59 |  |
| *Daniella oliveri* | 3.43±2.40 | 59.14±14.78 |  |
| *Diospyros mespiliformis* | 30.30±12.03 | 161.25±40.31 |  |
| *Lannea acida* | 159.98±57.20 | 398.84±99.71 |  |
| *Guiera senegalensis* | 1.43±1.43 | 8.62±2.16 |  |
| *Lannea velutina* | 5.70±3.93 | 6.95±1.74 |  |
| *Opilia celtifolia* | 1.22±1.22 |  |  |
| *Piliostigma thonningii* | 84.95±33.78 | 122.25±30.56 |  |
| *Prosopis africana* | 32.64±19.56 | 13.49±3.37 |  |
| *Pteleosis suberosa* | 25.03±9.93 | 74.92±18.73 |  |
| *Sarcocephalus latifolus* | 10.55±9.72 |  |  |
| *Securidaca longipedulata* | 0.63±0.63 | 3.86±0.96 |  |
| *Sterculia setigera* | 34.86±13.91 | 307.78±76.95 |  |
| *Terminalia laxiflora* | 105.73±25.29 | 120.11±30.03 |  |
| *Terminalia macroptera* | 9.39±5.31 | 49.27±12.32 |  |
| *Trichilia emetica* | 0.13±0.13 |  |  |
| *Xeroderris stulhmanii* | 1.07±1.07 |  |  |
| *Ximenia americana* | 1.56±1.56 | 12.76±3.19 |  |
| *Entada africana* | 54.23±29.85 |  |  |
| *Burkea africana* | 84.42±18.86 | 312.48±78.12 |  |
| *Anacardium occidentale* |  | 101.33±25.33 |  |
| *Azadirachta indica* |  | 87.41±21.85 |  |
| *Cassia siebiriana* | 14.94±7.15 | 25.58±6.39 |  |
| *Cassia singuana* |  | 76.78±19.19 |  |
| *Combretum fragrans* |  | 138.83±34.71 |  |
| *Combretum micranthum* |  | 99.63±24.91 |  |
| *Entada africana* |  | 67.80±16.95 |  |
| *Pseudocedrela kotschii* |  | 4.52±1.13 |  |
| *Strychnos innocua* |  | 26.07±6.52 |  |
| **Not preferred species** | | | |
| *Erythrina senegalensis* | 2.56±2.56 |  |  |
| *Feretia apodanthera* | 2.95±2.11 | 16.04±4.01 |  |
| *Hannoa undulata* | 1.08±0.74 |  |  |
| *Holarrhena floribunda* |  | 3.04 ± 0.76 |  |
| *Hymenocardia acida* | 0.16±0.16 |  |  |
| *Ficus glumosa* | 3.20±3.20 |  |  |
| *Ficus iteothylla* | 0.48±0.48 |  |  |
| *Ficus platiphyla* | 0.96±0.96 |  |  |
| *Ficus sur* | 0.62±0.62 |  |  |
| *Fluggea virosa* | 5.41±4.44 | 18.01±4.50 |  |
| *Grewia bicolor* | 1.85±1.60 |  |  |
| *Grewia mollis* | 10.45±4.60 |  |  |
| *Lonchocarpus laxiflorus* | 3.01±3.01 | 62.55±15.64 |  |
| *Maytenus senegalensis* | 8.20±4.07 | 20.21±5.05 |  |
| *Ozoroa insignis* | 1.29±1.29 | 6.52±1.63 |  |
| *Phyllanthus discoideus* | 1.39±1.39 |  |  |
| *Piliostigma reticulata* | 1.58±1.27 |  |  |
| *Stereospermum kunthianum* | 4.11±3.48 | 36.35±9.09 |  |
| *Strythnos innocua* | 2.78±2.46 |  |  |
| *Swartzia madagascarienis* | 2.11±1.65 | 74.99±18.75 |  |
| *Vitex diversifolia* | 2.06±2.06 |  |  |
| *Acacia pennata* | 3.58±3.58 |  |  |
| *Bridelia ferruginea* | 5.52±4.83 | 45.27±11.32 |  |
| *Cassia sieberiana* | 14.94±7.15 |  |  |
| *Cassia singuana* | 0.28±0.28 |  |  |
| *Dichrostachys cinerea* | 14.30±9.45 | 65.34±16.34 |  |
| *Hollarena floribunda* | 3.04±0.76 |  |  |
